# Effects of occupational exposures on respiratory health in steel factory workers

**DOI:** 10.3389/fpubh.2023.1082874

**Published:** 2023-02-14

**Authors:** Sajjad Mozaffari, Behzad Heibati, Maritta S. Jaakkola, Taina K. Lajunen, Safa Kalteh, Hadi Alimoradi, Mahsa Nazari, Ali Karimi, Jouni J. K. Jaakkola

**Affiliations:** ^1^Department of Occupational Health and Safety, School of Public Health, Tehran University of Medical Sciences, Tehran, Iran; ^2^Center for Environmental and Respiratory Health Research, Research Unit of Population Health, University of Oulu, Oulu, Finland; ^3^Biocenter Oulu, University of Oulu, Oulu, Finland; ^4^Department of Environmental Health Engineering, School of Public Health, Tehran University of Medical Sciences, Tehran, Iran; ^5^Esfahan Steel Company and Department of Occupational Health and Safety, Shahid Sadoughi University of Medical Sciences and Health Services, Yazd, Iran; ^6^Department of Occupational Health and Safety, Shahid Sadoughi University of Medical Sciences and Health Services, Yazd, Iran; ^7^Atmospheric Composition Research Unit, Finnish Meteorological Institute, Helsinki, Finland

**Keywords:** air quality, pulmonary function (PF), respiratory symptom, steel industry, epidemiological

## Abstract

**Background:**

The steel factory work environment contains various chemical exposures that can affect indoor air quality and have impact on respiratory health of the workers.

**Aims:**

The objective of this study was to assess potential effects of occupational exposures in steel factory workers in Iran on the respiratory symptoms, occurrence and the lung function levels.

**Method:**

This was a cross-sectional study of 133 men working in a steel factory forming the exposed group and 133 male office workers forming the reference group from a steel company in Iran. The participants filled in a questionnaire and underwent spirometry. Work history was used both as dichotomous (exposed/reference) and a quantitative measure of exposure, the latter measured as duration of exposure in the specified work (in years) for the exposed group and zero for the reference group.

**Results:**

Multiple linear regression and Poisson regression were used to adjust for confounding. In Poisson regression analyses, an increased prevalence ratio (PR) of all respiratory symptoms was observed in the exposed group. Lung function parameters were significantly reduced in the exposed group (*p* < 0.001). There was a dose–response relation between duration of occupational exposures and reduction in the predicted value of FEV1/FVC level (0.177, 95% CI −0.198 to −0.156) in all models.

**Conclusion:**

The results of these analyses showed that occupational exposures in steel factory work increase the prevalence of respiratory symptoms and reduce lung function. Safety training and workplace conditions were found to need improvement. In addition, use of proper personal protective equipment is recommended.

## Introduction

In steel industry, exposure to airborne contaminants is considered a risk factor for pulmonary diseases and for occurrence of pathological changes in airways (1, 2). Suspended metals, dust, and toxic gases in steelwork may increase the risk of respiratory symptoms related to diseases such as pneumoconiosis, bronchial asthma, COPD, and cancers (3, 4). Occupational exposure to harmful gases and fumes may cause a variety of respiratory outcomes, and these exposures may also lead to a significant decrease in pulmonary function, such as forced vital capacity (FVC), forced expiratory volume in one second (FEV1), and FEV1/FVC. Workers are often asymptomatic for a long time and may be diagnosed at a so late stage of the disease prevention or treatments are no longer effective (5). Dust particles are ubiquitous pollutants in the work environment of steel industry and are generated from iron ore, coke, and manganese processing steps. Potential association between occupational air pollutants, such as dust and heavy metals, and potential health effects has been a subject of research in several previous studies. For example, Soyseth et al. (6) reported that the prevalence of airflow limitation was higher in Norwegian exposed workers from smelters compared to non-exposed smelter employees. Chen et al. (7) reported that reduced lung function detected in Taiwanese steelworkers is partly due to exposure to inhalable dust particles and it can lead to reduction of both forced vital capacity (FVC) and forced expiratory volume in the first second (FEV1). Johnsen et al. (8) studied the relationship between occupational dust exposure and annual change in lung function among workers in 15 Norwegian smelters. Their results showed that in all smelters there was an annual decline in FEV1 in relation to increasing dust exposure (8). Johnsen et al. investigated potential impairment of lung function among workers from Norwegian smelters. They found that such impairment was significantly related to the job categories of line operator and non-line operator compared to the non-exposed employees (4). Søyseth et al. (9) found a significant association between occupational exposure and the incidence of airflow limitation in non-smoking workers from Norwegian smelters. Søyseth et al. (10) also investigated dust exposure and the incidence of work-related asthma-like symptoms (WASTH) in workers from Norwegian Smelters. They concluded that dust exposure was associated with an increased incidence of WASTH (10). Singh et al. (11) reported from India that the spirometric parameters including FVC, FEV1, FEV1/FVC ratio, FEF25–75, PEFR, PIFR, and FIVC were all significantly lower in an exposed group of steel workers compared to a reference group. Mousavian et al. (12) have reported exceeding air concentrations of Cr in a workplace from Iranian steel industry. In the study of Girma and Kebede (13) from Ethiopia, the FVC values of steel factory workers showed a strong negative correlation with duration of work and age of responders, and a weak negative correlation with the level of particulate matter (PM). Consistently with this, FEV1 values were strongly negatively correlated with exposure duration and the age of workers, while they were weakly negatively correlated with cross-sectional PM levels in the steel factory (13). Summarizing the literature, a significant reduction of ventilatory lung function and an increase in the occurrence of some respiratory symptoms, including prolonged cough, phlegm production, wheezing, bronchitis, shortness of breath, and bronchial asthma, as well as increased number of sick leaves have been found to be associated with long-term exposure to inorganic dust, fumes and toxic gases. However, to date, no study has been conducted on potential effects of occupational exposures of steel factory work on respiratory health among workers from the Middle Eastern countries, such as Iran. To fill in this gap in knowledge, the objectives of this study were to assess potential effects of occupational exposures in steel industry workers in Iran on the occurrence of respiratory symptoms and on the lung function level.

## Materials and methods

### Study design, and study population

This was a cross-sectional study of steel factory workers (the exposed group) and office workers (the reference group) from Iran. We invited 133 factory workers and 133 office workers from the same factory located in Isfahan. All individuals in both the exposed and unexposed groups (response rates 100%) participated in this study. The exposed group included general workers (*n* = 92), CO_2_ welding workers (*n* = 7), electric welding workers (*n* = 11), induction arc furnace workers (*n* = 13), and electric arc furnace workers (*n* = 10). Office workers were managers and other administrative staff, and the unexposed reference group also included chauffeurs and security staff. The sample size was based on a comparison of the prevalence of respiratory symptoms between the exposed and the reference group (beta = 90%, alpha = 0.05) (14).

### Data collection

The data collection comprised of three parts: air sampling, an interviewer-administered questionnaire and spirometry measurements. First, baseline information from the units with exposure emissions and subjects working there was collected. Information on respiratory symptoms, including current cough, phlegm production, cough with phlegm, wheezing, shortness of breath, and chest tightness were collected by using a standardized respiratory questionnaire translated and modified from the American Thoracic Society's (ATS) respiratory questionnaire (15). Data collection was based on this structured questionnaire and spirometric pulmonary functions tests (PFTs). The questionnaire also included information on (1) individual and working characteristics (age, gender, work experience, marital status, educational level, average working hours per day and per week), (2) history of respiratory diseases, and (3) smoking habits (as cigarettes/day). Participants were advised “If you are in doubt whether the answer is yes or no, answer no” (16). The pulmonary function tests for both groups were performed by the same technician at the workplaces. All participants gave written informed consent after being introduced to the nature of the study, potential risks and how their data may be used. The study was approved by the ethics committee of the Tehran University of Medical Sciences.

### Exposure assessment

The measurements and assessment of inhaled pollutants were performed as follows: The environmental and personal air sampling was carried out in 12 operation units (7 units sampled dust and 5 units fumes) to determine the pollutant concentrations and exposure levels of the workers. Calibrated personal sampling devices (manufactured by Casella, UK) were used to measure the concentrations of particles and of metal fumes at these stations in accordance with the OSHA guidelines. Dust samples were analyzed by gravimetric method, and fume samples were analyzed by atomic absorption spectrometry.

### Lung function

All the participants performed a spirometric lung function test with Fukuda Sangyo ST-150 spirometer (Fukuda, Japan). Forced vital capacity % predicted (FVC%) and forced expiratory volume in the first second % predicted (FEV1%) were already adjusted for sex, age, and height in the prediction equations. These were based on GLI spirometry reference values, i.e., multi-ethnic reference values for spirometry for the 3–95-year age range (17). FVC and FEV1 are the two essential parameters that measure ventilatory lung function. The accuracy and reproducibility of the test results by spirometry are affected by calibration of the device, operator experience, and effort of the patient to carry out the test correctly (18). In this study, the calibration and quality control of spirometry as well as performance of the flow volume measurements were carried out applying the guidelines of the American Thoracic Society and the European Respiratory Society Technical Statement (19). Lung function findings were then categorized into normal, obstruction, restriction, and combined obstruction with restriction.

### Statistical methods

Prevalence ratio (PR) was applied as the measure of effect of exposure on the risk of respiratory symptoms. We estimated adjusted PR's by Poisson regression analysis with logarithmic link function (SAS procedure GENMOD). We applied LSMEANS statement to obtain the effect estimates and their 95% CIs. The analyses of the respiratory symptoms were first adjusted for age and body mass index (BMI) (model 1), then additionally for smoking (model 2). Multiple linear regression was used to estimate the potential effects of occupational exposures on lung function levels. Work history was used as the quantitative measure of exposure, expressed as the duration of exposure in the specified work (in years) for the exposed group. For the reference group, the value of zero was given for this variable. The analyses of the absolute lung function levels (FEV1, FVC and FEV1/FVC) were first adjusted for age, height, and weight (model 1), and then additionally for smoking (model 2). We conducted sensitivity analyses by excluding smokers. We also used the Haldane-Anscombe correction by adding 0.5 to all cells for wheezing and chest tightness symptoms with zero observations in the unexposed group to be able to estimate the PRs and the 95% confidence intervals (CIs). Data analyses were performed applying SAS V.9.4 statistical package.

## Results

### Characteristics of the study population

The characteristics of the study population divided into the exposed group and the reference group are shown in Table 1. There were no statistically significant differences between the groups in terms of age, height, weight, BMI, or work history. Smoking was more common in the exposed group (*p* < 0.001).

**Table 1:** Characteristics of the study population including 133 steel factory workers and 133 office workers.

**Characteristic**	**Unexposed group (*n* = 133)**	**Exposed group (*n* = 133)**	***P*-value**
Age (year), mean ± SD	38.3 ± 5.0	39.13 ± 6.1	0.29^a^
Height (cm), mean ± SD	176.1 ± 5.0	175.4 ± 14.8	0.45^a^
Weight (kg), mean ± SD	82.4 ± 8.8	81.3 ± 11.7	0.38^a^
BMI (kg/m^2^), mean ± SD	26.7 ± 3.4	26.1 ± 3.6	0.20^a^
Work history (years), mean ± SD	13.0 ± 5.4	13.1 ± 5.1	0.73^a^
Smoking, *n* (%)^b^			<0.0001^b^
Never smoker	129 (97.0%)	82 (61.7%)	
Ex-smoker	0 (0.0%)	4 (3.0%)	
Current smoker	4 (3.0%)	47 (35.3%)	

^a^Independent samples t-test.

^b^χ^2^-test.

### Exposures

Table 2 shows the results of environmental measurements at the monitoring sites. The mean concentration of dust in six stations and the mean concentration of fumes (Fe and Mn) in two stations (conducting CO_2_ welding and electric welding) were above the corresponding acceptable exposure limit. The highest mean concentrations of dust were found in workshop 1 (wood dust, 4 mg/m^3^) and workshop 4 (casting sand, 0.83 mg/m^3^). The mean concentrations of dust in five workshops (wood and casting sand workshops 1,2,3,4 and 5) and the mean concentrations of fumes in CO_2_ welding (Fe and Mn) and Electric welding (Fe) exceeded the designated acceptable exposure limits (20). The levels of dust in workshops 2–5 were 7.6, 16.2, 16.6, and 10.6 times higher than the acceptable exposure limits (Table 2). In the CO_2_ welding unit, the levels of Fe fumes slightly exceeded the maximum allowable limit, while the levels of Mn fumes were 2.7 times greater than the acceptable limit. In addition, the Fe fumes in electric welding unit were slightly greater than the permissible exposure limit.

**Table 2:** Concentrations of dusts and fumes measured in 11 units of the company.

**Station**	**Workshop**	**Pollutant**		**Mean concentration (mg/m^3^)**	**Permissible exposure limit**
1	Workshop 1	Dust	Wood	**4**	0.5
2	Workshop 2		Casting sand	**0.38**	
3	Workshop 3		Casting sand	**0.81**	
4	Workshop 4		Casting sand	**0.83**	
5	Workshop 5		Casting sand	**0.53**	
6	Workshop 6		Casting sand	0.40	
7	Workshop 7		Casting sand	0.48	
8	CO_2_ welding	Fume	(AS Fe_2_O_3_) Fe	**5.96**	5
			(AS Mn) Mn	**0.54**	0.2
			(AS Cu) Cu	0.002	0.2
			(AS Zno) Zn	0.003	2
			(AS Pb) Pb	0.00	0.05
9	Electric welding	Fume	(AS Fe_2_O_3_) Fe	**5.85**	5
			(AS Mn) Mn	0.14	0.2
			(AS Cu) Cu	0.001	0.2
			(AS Zno) Zn	0.01	2
			(AS Pb) Pb	0.00	0.05
10	Induction Furnace	Fume	(AS Fe_2_O_3_) Fe	0.53	5
			(AS Mn) Mn	0.001	0.2
			(AS Cu) Cu	0.001	0.2
			(AS Zno) Zn	0.001	2
			(AS Pb) Pb	0.00	0.05
11	Electric arc furnace	Fume	(AS Fe_2_O_3_) Fe	3.15	5
			(AS Mn) Mn	0.06	0.2
			(AS Cu) Cu	0.001	0.2
			(AS ZnO) Zn	0.01	2
			(AS Pb) Pb	0.00	0.05

Bold values indicate the statistically significant effects.

### Prevalence of lung function impairment

**Figure 1:**
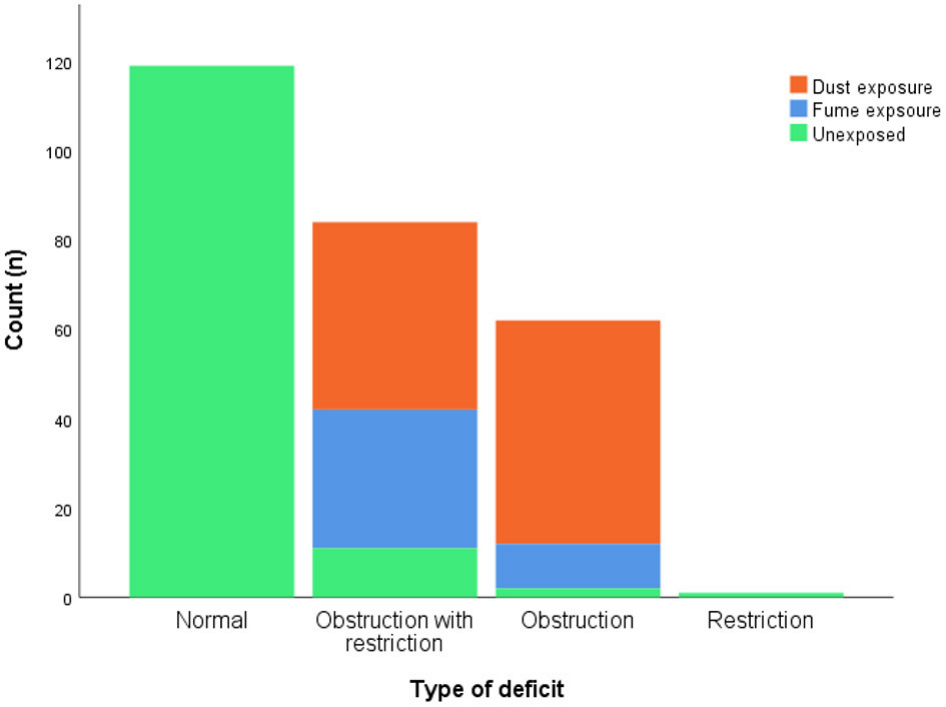
Prevalence of lung function deficits.

Figure 1 and Supplementary Table S1 show the prevalence of lung function deficits in the two categories of both groups. The overall prevalence of lung function impairment was 100% (*n* = 113) in the exposed group, while it was 10.5% (*n* = 14) in the reference group. In the exposed group, the majority of those with lung function impairment had a combination of obstruction with restriction (54.9%), followed by obstruction alone (45.1%). In addition, obstruction with restriction (75.6%) and obstruction alone (54.4%) were found to be the major impairments among those who were exposed to fumes and dust, respectively.

### Effects of exposure on respiratory symptoms

The prevalence of respiratory symptoms in the exposed and reference groups is presented in Table 3. The exposed group showed consistently a higher prevalence of all respiratory symptoms. In the fully adjusted models, the PR (95% CI) was 20.20 (7.0–58.39) for cough, 24.01 (7.19–80.20) for phlegm, 24.30 (7.48–78.83) for cough with phlegm, and 15.93 (4.84–52.40) for shortness of breath. In addition, the PRs of respiratory symptoms related to fume and dust exposures, when these were evaluated separately, were statistically significantly increased in the exposed group compared to the reference group (Table 3).

**Table 3:** Prevalence of respiratory symptoms among steel factory workers compared to the reference group.

	**Unexposed group (*n* = 133) *n* (%)**	**Exposed (*n* = 133) *n* (%)**	**Unadjusted model PR (95% CI)**	**Adjusted model 1^a^ PR (95% CI)**	**Adjusted model 2^b^ PR (95% CI)**
**Exposure (reference** = **0, exposed group** = **1)**					
**Symptom**					
Cough	4 (3.0)	64 (48.5)	**16.12 (6.05 to 42.98)**	**16.46 (6.21 to 43.62)**	**20.20 (7.0 to 58.39)**
Phlegm	3 (2.3)	57 (42.9)	**19.0 (6.10 to 59.20)**	**20.28 (6.60 to 62.31)**	**24.01 (7.19 to 80.20)**
Cough with phlegm	3 (2.3)	85 (63.9)	**28.33 (9.19 to 87.34)**	**28.20 (9.21 to 86.36)**	**24.30 (7.48 to 78.83)**
Wheezing^c^	0 (0)	50 (37.6)	**101.0 (6.29 to 1620.20)**		
Shortness of breath	3 (2.3)	73 (54.9)	**24.33 (7.88 to 75.19)**	**23.70 (7.73 to 72.66)**	**15.93 (4.84 to 52.40)**
Chest tightness ^c^	0 (0)	35 (26.3)	**71.0 (4.40 to 1145.56)**		
**Exposure (reference** = **0, dust exposure** = **1)**					
Symptom	Reference group (*n* = 133) *n* (%)	Exposed (*n* = 92) *n* (%)			
Cough	4 (3.0)	45 (48.9)	**16.44 (6.13 to 44.13)**	**16.72 (6.29 to 44.47)**	**20.15 (6.80 to 59.70)**
Phlegm	3 (2.3)	41 (44.6)	**19.76 (6.30 to 61.93)**	**20.94 (6.78 to 64.69)**	**24.44 (7.11 to 84.05)**
Cough with phlegm	3 (2.3)	58 (63.0)	**27.95 (9.03 to 86.48)**	**27.80 (9.08 to 85.07)**	**24.65 (7.43 to 81.72)**
Wheezing^c^	0 (0)	36 (39.1)	**105.18 (6.53 to 1692.33)**		
Shortness of breath	3 (2.3)	51 (55.4)	**24.58 (7.91 to 76.32)**	**23.65 (7.71 to 72.57)**	**16.51 (4.87 to 55.93)**
Chest tightness^c^	0 (0)	22 (23.9)	**64.83 (3.98 to 1055.56)**		
**Exposure (reference** = **0, fume exposure** = **1)**					
Symptom	Reference group (*n* = 133) *n* (%)	Exposed (*n* = 41)			
Cough	4 (3.0)	19 (46.3)	**15.41 (5.56 to 42.67)**	**15.28 (5.56 to 42.03)**	**13.18 (3.28 to 53.0)**
Phlegm	3 (2.3)	16 (39.0)	**17.30 (5.30 to 56.47)**	**18.05 (5.85 to 55.74)**	**15.96 (4.34 to 58.73)**
Cough with phlegm	3 (2.3)	27 (65.9)	**29.20 (9.34 to 91.26)**	**29.23 (9.50 to 90.02)**	**17.0 (4.68 to 61.78)**
Wheezing^c^	0 (0)	14 (34.2)	**92.52 (5.63 to 1518.18)**		
Shortness of breath	3 (2.3)	22 (53.7)	**23.79 (7.52 to 75.27)**	**24.21 (7.82 to 74.93)**	**8.60 (1.75 to 42.15)**
Chest tightness^c^	0 (0)	13 (31.7)	**86.14 (5.23 to 1418.44)**		

The crude and adjusted prevalence ratios (PRs) are from Poisson regression models.

Statistically significant effects are shown as bolded.

^a^Adjusted for age, BMI.

^b^Adjusted for age, BMI, smoking (current, former, passive).

^c^Haldane-Anscombe corrected PR.

### Effects of exposure on lung function

Table 4 shows the effects of the steel work exposure on the absolute pulmonary function levels and on %-predicted measures. The absolute FEV1, FVC, and FEV1/FVC levels were reduced among the exposed group compared to the reference group even after adjusting for confounders, including smoking. A dose–response pattern was observed for the associations between work duration and lung function levels. In the unadjusted model, there was a significant reduction in the absolute and the %-predicted FEV1 for each year of increasing work duration. Statistically significant reduction in FEV1/FVC % predicted was detected per year of exposure duration in all models. In addition, the levels of all absolute pulmonary function parameters were lower among workers exposed to both dust and fumes (when these were evaluated separately) and compared to the office workers.

**Table 4:** Effects on lung function parameters in relation to any exposure, duration of exposure and exposure to fumes and dusts separately.

	**Unadjusted model**	**Adjusted model 1 beta (95% CI)**	**Adjusted model 2 beta (95% CI)**
**Exposure (reference** = **0, exposed group** = **1)**			
**Parameter**			
FEV_1_, L (effect of the exposure)	**−0.677 (−0.817 to** **−0.538)**	**−0.672 (−0.790 to** **−0.555)**	**−0.634 (−0.767 to** **−0.506)**
FVC, L (effect of the exposure)	**−0.695 (−0.881 to** **−0.510)**	**−0.702 (−0.861 to** **−0.544)**	**−0.662 (−0.838 to** **−0.485)**
FEV_1_/FVC (effect of the exposure)	**−0.035 (−0.045 to** **−0.024)**	**−0.033 (−0.044 to** **−0.023)**	**−0.030 (−0.042 to** **−0.018)**
FEV1, % predicted (effect of the exposure)	**–**0.053 (**–**0.174 to 0.067)		0.018 (**–**0.115 to 0.151)
FVC, % predicted (effect of the exposure)	**–**0.063 (**–**0.223 to 0.097)		0.030 (**–**0.150 to 0.207)
FEV1/FVC, % predicted (effect of the exposure)	**–**0.153 (**–**0.396 to 0.090)		**–**0.078 (**–**0.348 to 0.192)
**Effect of duration of exposure (reference**=**0, exposed group**=**1)**			
FEV_1_, L (change per year of exposure)	**−0.025 (−0.045 to** **−0.005)**	0.016 (**–**0.014 to 0.046)	0.017 (**–**0.012 to 0.047)
FVC, L (change per year of exposure)	**–**0.026 (**–**0.054 to 0.001)	0.023 (**–**0.020 to 0.066)	0.025 (**–**0.018 to 0.068)
FEV_1_/FVC (change per year of exposure)	**–**0.001 (**–**0.002 to 0.0004)	0.0002 (**–**0.002 to 0.002)	0.0003 (**–**0.001 to 0.002)
FEV1, % predicted (change per year of exposure)	**−0.024 (−0.047 to** **−0.002)**		**–**0.022 (**–**0.044 to 0.0001)
FVC, % predicted (change per year of exposure)	**–**0.021 (**–**0.051 to 0.009)		**–**0.018 (**–**0.048 to 0.012)
FEV1/FVC, % predicted (change per year of exposure)	**−0.178 (−0.199 to** **−0.157)**		**−0.177 (−0.198 to** **−0.156)**
**Exposure (reference**=**0, fume exposure**=**1)**			
FEV1, L (effect of the exposure)	**−0.630 (−0.864 to** **−0.396)**	**−0.691 (−0.895 to** **−0.487)**	**−0.580 (−0.826 to** **−0.336)**
FVC, L (effect of the exposure)	**−0.640 (−0.955 to** **−0.325)**	**−0.728 (−1.005 to** **−0.449)**	**−0.577 (−0.911 to** **−0.244)**
FEV1/FVC (effect of the exposure)	**−0.039 (−0.057 to** **−0.022)**	**−0.038 (−0.056 to** **−0.021)**	**−0.032 (−0.053 to** **−0.012)**
FEV1, % predicted (effect of the exposure)	**–**0.109 (**–**0.314 to 0.095)		0.063 (**–**0.176 to 0.303)
FVC, % predicted (effect of the exposure)	**–**0.151 (**–**0.425 to 0.123)		0.067 (**–**0.255 to 0.390)
FEV1/FVC, % predicted (effect of the exposure)	0.028 (**–**0.325 to 0.381)		0.294 (**–**0.118 to 0.707)
**Exposure (reference**=**0, dust exposure**=**1)**			
FEV1, L (effect of the exposure)	**−0.699 (−0.828 to** **−0.569)**	**−0.663 (−0.767 to** **−0.558)**	**−0.666 (−0.781 to** **−0.551)**
FVC, L (effect of the exposure)	**−0.720 (−0.883 to** **−0.558)**	**−0.691 (−0.820 to** **−0.562)**	**−0.706 (−0.850 to** **−0.563)**
FEV1/FVC (effect of the exposure)	**−0.033 (−0.045 to** **−0.021)**	**−0.030 (−0.042 to** **−0.018)**	**−0.028 (−0.041 to** **−0.015)**
FEV1, % predicted (effect of the exposure)	**–**0.029 (**–**0.099 to 0.042)		0.008 (**–**0.087 to 0.070)
FVC, % predicted (effect of the exposure)	**–**0.023 (**–**0.112 to 0.065)		0.005 (**–**0.092 to 0.103)
FEV1/FVC, % predicted (effect of the exposure)	**–**0.234 (**–**0.489 to 0.022)		**–**0.253 (**–**0.536 to 0.031)

Statistically significant effects are shown as bolded.

Model 1: Adjusted for age (for the actual values), height (for the actual values), and weight (for the actual values).

Model 2: Adjusted for age (for the actual values), height (for the actual values), weight (for the actual values), and smoking (current, former, and passive).

## Discussion

In this cross-sectional study, the prevalence of respiratory symptoms and level of lung function parameters were compared between steel factory workers and office workers from the same steel industry. We found that exposed study subjects experienced increased prevalence of all respiratory symptoms. Furthermore, the prevalences of obstruction, and obstruction in combination with restriction were higher in the exposed group (Supplementary Table S1). We also observed significant reductions in the absolute pulmonary function levels in the exposed group, and when addressing different types of steel work exposures separately, among those exposed to either fumes or dust when compared to the reference group. Both the absolute and %-predicted values of FEV1 were significantly related to the duration of steel factory exposure in the unadjusted model. Predicted FEV1/FVC% was significantly related to the exposure duration even after adjustment for confounding factors. The results of the sensitivity analyses indicated similar effect estimates (data not shown).

### Validity of the results

All individuals in both groups (response rates 100%) participated in this study. The characteristics of the study population, apart from smoking habits, were not statistically significantly different between the compared groups. This was a cross-sectional study, so it was not feasible to elaborate on the possibility that workers with respiratory health problems may have been more likely to leave work compared to workers who remained healthy. This type of selection bias would lead to underestimation of the relations of interest, i.e., our estimates could underestimate the real effects. We collected information on several characteristics of the study population and adjusted the analyses of respiratory symptoms and lung function for personal characteristics (BMI, age) and for smoking. Therefore, we believe that confounders are not a likely explanation for our findings.

### Synthesis with previous knowledge

We found increased occurrence of respiratory symptoms and reduced spirometric lung function parameters among steel factory workers. Our results are in line with some prior studies conducted in steel industry as well as in other industries. Sobaszek et al. (21) reported acute respiratory effects of welding fumes in the workplace by measuring the across-shift changes in a population of 144 stainless steel (SS) and mild steel (MS) welders and 223 controls. A significant decrease in forced vital capacity (FVC) during the shift was observed. Moreover, the across-shift decreases in FEV1, FVC, and peak expiratory flow (PEF) were significantly related to the Manual Metal Arc welding process, compared with Metal Inert Gas techniques (PEF = −2.7% of baseline values (SD, 11.9) vs. 2.0% of baseline values (SD, 7.7) *P* = 0.04; FVC = −1.5% of baseline values (SD, 4.8) vs. 0.2% of baseline values (SD, 4.5) *P* = 0.05). A significant influence of the duration of stainless-steel welding exposure on the change of lung function during the work shift was reported. No significant differences between smokers, ex-smokers, and never-smokers were found in terms of the change of ventilatory function during the work shift. The authors concluded that welding-related lung function reductions are seen in stainless-steel welders compared with mild steel welders, as well as in those with a longer lifetime welding history. Giahi et al. (1) examined FVC, FEV1, and FEV1/FVC levels between the exposed and the reference groups in a steel plant from Iran. Their analysis showed that FEV1/FVC values were statistically significantly lower in the exposed group (1). Another study by Koo et al. (22) from South Korea investigated potential effect of silica dust on pulmonary function levels in foundry workers. They found that excess exposure to silica dust among the exposed workers was associated with lower lung function levels, apart from FVC levels, suggesting obstructive pattern (23). Gholami et al. (24) from Iran reported statistically significantly reduced levels of pulmonary function parameters (including FVC, FEV1, FEV1/ FVC, and PEF) in the exposed compared to non-exposed workers at an iron-ore mine in eastern Iran (23). Several other studies have also associated increased dust levels with decreased pulmonary function levels in different types of industries, including brick manufacturing, tile and ceramic industry, and steel industry (7, 13, 24–26).

In our study, all respiratory symptoms were more prevalent in the exposed group compared to the reference group. Sakar et al. (27) found cough and mucus as the most prevalent symptoms among ceramic workers from Turkey. Other studies from Iran have indicated that occupational exposure to excessive levels of dust leads to respiratory symptoms, including cough, mucus production and shortness of breath (1, 23). Similar findings have been reported from other studies conducted in different types of industries, including stoneworkers, miners, and cement factory workers (23, 28). In the present study, we found that the dust levels exceeded the acceptable limit in most of the workstations. We showed that occupational exposure to indoor pollutants in a steel factory laeds to increased respiratory symptoms in the exposed workers compared to office workers. In addition, our study showed a dose-response effect of steel work exposure on reduction of FEV1 (both absolute level and %-predicted). The predicted value of FEV1/FVC% was also reduced significantly in relation to the duration of exposure even after adjustment for confounding. This finding is consistent with the studies conducted in Tanzania and Iran. Mwaiselage et al. (28) studied potential effects of cement dust exposure on ventilatory lung function. They found an increased odds ratio of 9.9 (95% CI: 3.5–27.6) for airflow limitation among workers with cumulative total dust exposure of >300.0 mg/m^3^ compared to workers with cumulative total dust exposure of <100.0 mg/m^3^ year. This indicated that workers with long-term exposure to high cement dust levels experienced an excess risk of developing airflow limitation (28). Gholami et al. (24) reported an association (OR: 1.155, CI: 1–1.333) between respirable dust exposure among iron ore mine workers and increased occurrence of shortness of breath (23). Aminian et al. (29) found an association between exposure to workplace fumes and dust and shortness of breath (OR: 4.55, CI: 1.66–12.45) among workers of a steel galvanization factory in Arak, Iran. Consistently with previous studies, our results showed that workers from a steel factory experience an increased risk of respiratory symptoms. Furthermore, our study found signs of reduced FEV1 and FEV1/FVC, which is consistent with obstructive lung disease. In addition, some workers had reduced FVC levels, which suggests restrictive lung function impairment in relation to exposure to dusts in our study. Reduced FEV1 and FEV1/FVC levels were associated with years of exposure in the study by Meo et al. (30), which was in line with our findings.

As the work-related adverse respiratory effects among steel factory workers are likely to be mostly preventable, we recommend that primary preventive actions should be conducted whenever it is possible. As we found in this study, steel factory workplaces still have not taken widely into use basic precautionary measures, such as training on how to protect against exposures present at such factory work or on how to recognize if there is a need for respiratory protection. Such training should also include information on the type of protective equipment that should be used when harmful exposures cannot be avoided in other ways. Another important task is to provide training on how to use protective masks in appropriate way, especially when it is not possible to provide specific local ventilation. For example, N95 respirators recommended by the US National Occupational Safety and Health Administration (OSHA) would provide protection against many particulates but they provide little protection against vapors or gasses (20).

We assume that the environmental conditions and the workers of the studied factory represent well the situation in other factories in Iran and other countries in the same region and thus, the results are generalizable to factory workplaces in Middle Eastern countries and to countries with similar production and standards of living.

## Conclusions

This study investigated the relations between occupational exposures in a steel factory and occurrence of respiratory symptoms and lung function deficits in workers of this industry. Our study provides evidence that exposure to dust and fumes in steel industry is related to significantly increased occurrence of respiratory symptoms, and to significant spirometric lung function deficits, including both restriction and obstruction. A dose–response relation was shown between years of employment in steel industry and reduced level of both absolute and %-predicted FEV1. Thus, these results showed that the exposed workers in steel industry are at a high risk of experiencing several respiratory symptoms and lung function impairment. Taking into use appropriate protective respiratory equipment and application of efficient local exhaust system and ventilation is recommended in the future for the steel industry to protect the workers' health.

## Data availability statement

The raw data supporting the conclusions of this article will be made available by the authors, without undue reservation.

## Ethics statement

The studies involving human participants were reviewed and approved by the Ethics Committee of the Tehran University of Medical Sciences. The patients/participants provided their written informed consent to participate in this study.

## Author contributions

Data were collected by SM, SK, HA, and MN. Analysis was performed by TL and BH. The manuscript was drafted by BH, MJ, and SM. Reviewed by MJ and supervised with AK and JJ. SM and BH contributed equally to this paper. All authors were involved in editing of the manuscript for intellectual content.
