# Supplementary material for: Effects of occupational exposures on respiratory health in steel factory workers
Source: Front Public Health. 2023 Feb 14;11:1082874. doi: 10.3389/fpubh.2023.1082874 (PMC9971494; doi:10.3389/fpubh.2023.1082874)
Supplement: Supplementary file 1 [file Table_1.pdf]

Table S1 Prevalence of lung function deficits

Type of deficit	Unexposed group (n=133)	Exposed group (n=133)	Type of exposure	
			Dust exposure (n=92)	Fume exposure (n=41)
Obstruction [a]	2 (1.5%)	60 (45.1%)	50 (54.4%)	10 (24.4%)
Restriction [b]	1 (0.8%)	0 (0%)	0 (0%)	0 (0%)
Obstruction with restriction [c]	11 (8.3%)	73 (54.9%)	42 (45.6%)	31 (75.6%)
Normal [d]	119 (89.4%)	0 (0%)	0 (0%)	0 (0%)

[a] FVC normal (≥80%), FEV_1 reduced (<80%), FEV_1/FVC reduced (<0.7).
[b] FVC reduced (<80%), FEV_1 normal or reduced (≥80% or <80%), FEV_1/FVC normal or increased (≥0.7).
[c] FVC reduced (<80%), FEV_1 reduced (<80%), FEV_1/FVC reduced (<0.7).
[d] FVC normal (≥80%), FEV_1 normal (≥80%), FEV_1/FVC normal (≥0.7).
